# A Systematic Review and Meta-analysis of Neuroimaging in Oppositional Defiant Disorder (ODD) and Conduct Disorder (CD) Taking Attention-Deficit Hyperactivity Disorder (ADHD) Into Account

**DOI:** 10.1007/s11065-015-9315-8

**Published:** 2016-02-05

**Authors:** Siri D. S. Noordermeer, Marjolein Luman, Jaap Oosterlaan

**Affiliations:** Faculty of Behavioural and Movement Sciences, Clinical Neuropsychology Section, VU University Amsterdam, Van der Boechorststraat 1, 1081 BT Amsterdam, The Netherlands

**Keywords:** ODD, CD, ADHD, Structural MRI, Functional MRI, ALE meta-analysis

## Abstract

Oppositional defiant disorder (ODD) and conduct disorder (CD) are common behavioural disorders in childhood and adolescence and are associated with brain abnormalities. This systematic review and meta-analysis investigates structural (sMRI) and functional MRI (fMRI) findings in individuals with ODD/CD with and without attention-deficit hyperactivity disorder (ADHD). Online databases were searched for controlled studies, resulting in 12 sMRI and 17 fMRI studies. In line with current models on ODD/CD, studies were classified in hot and cool executive functioning (EF). Both the meta-analytic and narrative reviews showed evidence of smaller brain structures and lower brain activity in individuals with ODD/CD in mainly hot EF-related areas: bilateral amygdala, bilateral insula, right striatum, left medial/superior frontal gyrus, and left precuneus. Evidence was present in both structural and functional studies, and irrespective of the presence of ADHD comorbidity. There is strong evidence that abnormalities in the amygdala are specific for ODD/CD as compared to ADHD, and correlational studies further support the association between abnormalities in the amygdala and ODD/CD symptoms. Besides the left precuneus, there was no evidence for abnormalities in typical cool EF related structures, such as the cerebellum and dorsolateral prefrontal cortex. Resulting areas are associated with emotion-processing, error-monitoring, problem-solving and self-control; areas associated with neurocognitive and behavioural deficits implicated in ODD/CD. Our findings confirm the involvement of hot, and to a smaller extent cool, EF associated brain areas in ODD/CD, and support an integrated model for ODD/CD (e.g. Blair, *Development and Psychopathology, 17*(3), 865-891, 2005).

## Introduction

In recent years, magnetic resonance imaging (MRI) studies have provided insight into the underlying brain mechanisms of disruptive behaviour disorders, including oppositional defiant disorder (ODD) and conduct disorder (CD). This review will integrate and discuss studies using structural (sMRI) and functional MRI (fMRI) in these disorders. ODD and CD are developmental disorders that are among the most commonly diagnosed mental health conditions in childhood (Hamilton and Armando 2008; Loeber et al. 2009). Community samples show a prevalence rate for ODD ranging between 2 and 14 % and for CD ranging between 2 and 16 % (Boylan et al. 2007; Loeber et al. 2000). Both disorders are more prevalent in boys than in girls with ratio’s ranging from 3:1 to 9:1 (Loeber et al. 2000). ODD is defined by a frequent and persistent pattern of irritable and angry mood, vindictiveness and developmentally inappropriate, negativistic, defiant, and disobedient behaviour toward authority figures (American Psychiatric Association 2013). CD is characterised by a persistent pattern of multiple antisocial behaviours during childhood and adolescence, including fighting, bullying, stealing, vandalism, and lying for personal gain (American Psychiatric Association 2013). Depending on whether the individual was younger or older than 10 years at the time of symptom onset, there is a differentiation between childhood-onset CD and adolescent-onset CD (American Psychiatric Association 2013).

Although both disorders have some distinct characteristics, the general consensus is that ODD and CD are highly correlated expressions of psychopathology. Till the emergence of the DSM-5, ODD has been coined as a milder version of CD as emphasised by the hierarchical rule stipulated in the DSM-IV stating that a diagnosis of ODD is precluded when CD is present, due to very high levels of ODD features in individuals with CD and the precursory role of ODD for CD. This precursory role of ODD for the later development of CD is supported by a quadrupled risk for the development of CD in individuals with ODD (Burke et al. 2002; Loeber et al. 2009; Rowe et al. 2010). In addition to the high levels of ODD features in CD, comorbidity rates of ODD are as high as 45 % in children with CD, and in clinical samples these rates increase to up to 96 % (Loeber et al. 2009; Rowe et al. 2002). ODD and CD share risk factors in both the psychosocial domain, such as poverty and social disadvantage, and the family domain, such as a history of criminality in biological parents (Rowe et al. 2002; Burke et al. 2002). An extensive review on the heritability of ODD and CD shows heritability rates of 61 and 74 %, respectively, with 50 % of the reported genes being associated with both disorders (Coolidge et al. 2000; Lahey and Waldman 2012). The presence of either ODD or CD predicts poor future outcomes, including compromised psychiatric, family and social functioning, as well as an increased risk for adverse life events: e.g., peer rejection, criminal behaviour and incarceration at a young age (Burke et al. 2002; Hamilton and Armando 2008; Loeber et al. 2000). When ODD or CD persists, individuals are at a heightened risk for anxiety disorders and depression. Furthermore, persistence of childhood ODD or CD into adulthood results in a diagnosis of antisocial personality disorder (APD), which is in turn related to high rates of domestic violence, unemployment and homelessness (Loeber et al. 2008; Kimonis and Frick 2010). APD can only be diagnosed when there is a history of some symptoms of CD and the transition from CD to APD occurs in around 54 % of individuals with CD (Fairchild et al. 2013b; American Psychiatric Association 2013). Treatment of ODD and CD is generally not specific to either disorder, and a combination of interventions that aim at multiple domains tends to be more successful than treating a singular domain. In the current review, ODD and CD will be treated as representing one dimension of psychopathology, because of the similarities in many domains including aetiology, phenotypical manifestation, correlated features, as well as treatment. We will refer to this dimension as ODD/CD.

Several explanatory models of ODD/CD focus specifically on neurocognitive impairments, which are thought to be related to abnormalities in underlying brain mechanisms. Neurocognitive impairments that are associated with ODD/CD include lower IQ, deficiencies in inhibitory control, abnormalities in emotional processing and social cognition, and abnormalities in reinforcement processing. Most of the explanatory models emphasise a deficit in so-called executive functioning (EF). EF is the sum of neurocognitive processes that maintain an appropriate problem-solving set to attain a goal (Pennington and Ozonoff 1996; Willcutt et al. 2005). A well-known distinction in EF is that between hot and cool EF. Hot EF is characterised by motivational and affective aspects of cognitive processing, such as reinforcement learning, affective decision-making and emotional processing (Anderson et al. 2008; Blair and Lee 2013; Kerr and Zelazo 2004; Zelazo and Carlson 2012). Brain areas that are reported to be important for hot EF include the amygdala, anterior cingulate cortex, insula and orbitofrontal cortex (Crowe and Blair 2008; Prencipe et al. 2011; Rubia 2011). In contrast, cool EF refers to goal-directed and problem-solving behaviours, as well as self-regulation, not involving motivational or affective aspects. Cool EF encompasses functions using diverse abilities such as inhibition, working memory, planning, flexibility, and the ability to creatively generate solutions for problems (Sarkar et al. 2013; Diamond 2013). Brain areas reported to be central to cool EF include the dorsolateral prefrontal cortex and the cerebellum (Prencipe et al. 2011; Rubia 2011; Yang and Raine 2009; Sterzer and Stadler 2009). This distinction between hot and cool EF provides a framework to study underlying brain mechanisms of observed behavioural and neurocognitive abnormalities in ODD/CD. This knowledge can help test theoretical models on ODD/CD through clarifying involvement of brain areas central to those models. This important information on theoretical model building can in turn help to further advance the field, by yielding supporting or opposing evidence for the involvement of brain areas.

An altered reinforcement system, and thus a hot EF problem, was proposed in early models on ODD/CD by Quay (1965, 1993) and by Newman and Kosson (1986). Indeed, a recent extensive review in antisocial individuals showed altered sensitivity to reward and punishment and processing of these contingencies (Byrd et al. 2014). Specifically, antisocial individuals show an increased affinity for immediate reward over delayed reward, an insensitivity to punishment, and increased reward-seeking (Byrd et al. 2014). This altered reinforcement sensitivity has been related to problems in social cognition in individuals with ODD/CD. For example, these individuals tend to show a preference for more aggressive reactions in social situations, which might be due to their unsparing surge for reward and decreased punishment sensitivity (Burke et al. 2002; Loeber et al. 2008; Quay and Hogan 1999; Rubia 2011). In addition, abnormalities in emotional processing have been reported repeatedly in studies with ODD/CD samples (Byrd et al. 2014), including reduced levels of empathy and deficits in the recognition of emotional expressions (Blair 2013). Both abnormalities in reinforcement sensitivity and emotional processing in ODD/CD have been related to abnormalities in hot EF brain areas, such as the amygdala and the striatum (Crowe and Blair 2008; Prencipe et al. 2011).

Another important explanatory model, that is more fitting with a cool EF deficit, was proposed by Moffitt (1993), who distinguished between adolescent-limited ODD/CD, and the more severe, life-course persistent ODD/CD (Moffitt 1993). According to that model, adolescent-limited ODD/CD is merely a stage in development during which adaptive social behaviour is tested and learned, while life-course persistent ODD/CD is thought to arise of an interplay between a difficult and under-controlled temperament and adverse environmental factors. This under-controlled temperament is thought to be promoted by a deficit in cool EF, including difficulties in inhibition and self-control (Burke et al. 2002; Loeber et al. 2008; Moffitt 1993). Evidence from neurocognitive studies generally points toward a range of abnormalities in cool EF in ODD/CD, such as low IQ, inefficiencies in problem solving, and less than optimal inhibitory control (Oosterlaan et al. 1998; Burke et al. 2002; Loeber et al. 2008; Quay and Hogan 1999, but see Van Goozen et al. 2004), and have been related to abnormalities in the dorsolateral prefrontal cortex and cerebellum (Yang and Raine 2009; Prencipe et al. 2011).

Finally, one of the most influential explanatory models at present is proposed by Blair, who suggests that individuals with ODD/CD demonstrate impairments in two separate circuits associated with hot and cool EF (Blair 2005). According to Blair (2005), the first compromised circuit is involved in emotional processing and regulation and is responsible for an increase in antisocial behaviour. The key component of brain areas underlying this mainly hot EF circuit is supposedly the amygdala. The second compromised circuit is involved in response inhibition and is responsible for loss of temper and exaggerated aggressive responses in individuals with ODD/CD. The key component of brain areas underlying this mainly cool EF circuit is supposedly the ventrolateral frontal cortex.

Support for the explanatory models on ODD/CD is well established in behavioural studies. However, knowledge about these models in terms of structural and functional neuroimaging is incomplete. While a review of the current structural and functional neuroimaging literature would be an ideal way to provide neurobiological evidence to confirm or reject a model, such a review has not been conducted so far. The current review aims to fill this gap with the goal to enhance insight into the underlying mechanisms of ODD/CD and additionally test the plausibility of hot and cool EF models in ODD/CD in terms of neural mechanisms.

A highly comorbid condition of ODD/CD is attention-deficit hyperactivity disorder (ADHD), one of the most commonly diagnosed disruptive behaviour disorders in children. The percentage of individuals diagnosed with ODD/CD that additionally qualify for a comorbid ADHD diagnosis ranges up to 35 %, and up to 50 % of children with disruptive behaviours show symptoms of both ADHD and ODD/CD (Loeber et al. 2000; Anderson and Kiehl 2013; Connor et al. 2010; Waschbusch 2002). In children and adolescents with ODD/CD and comorbid ADHD, the prognosis, including the risk to develop anxiety and depressive disorders and antisocial personality disorder, is considerably worse than when only ODD/CD or only ADHD is present (Dolan and Lennox 2013; Loeber et al. 2000). In addition, this comorbid group shows an earlier age of symptom onset, exhibits more physical aggression and delinquency, shows significantly higher ODD, CD and ADHD symptom severity, and shows more functional impairments than a group with any of these diagnoses in singularity (Anderson and Kiehl 2012; Loeber et al. 2000; Waschbusch 2002). This highlights the importance of clarifying the specificity of abnormalities associated with ODD/CD and contrasting ODD/CD-only with ODD/CD+ADHD, when studying these disorders.

Although previous reviews have addressed structural and functional brain abnormalities in ODD/CD (Matthys et al. 2013; Rubia 2011), these reviews were neither systematic reviews, nor focussed exclusively on diagnostic groups of ODD/CD, nor were set out to investigate the relative contribution of ADHD. To address structural and functional brain anatomical aspects of ODD/CD, the current comprehensive review includes a complete systematic narrative review as well as meta-analyses using ALE (Eickhoff et al. 2009, 2012; Laird et al. 2005) of the available structural and functional imaging studies. The quantitative approach increases the precision and the power of reported results compared to a purely qualitative review, and indicates which brain areas are most robustly implicated in ODD/CD. However, since an ALE meta-analysis does not allow inclusion of studies reporting on non-significant group differences, the narrative review provides the necessary balance in interpreting the findings. The narrative review complied with the standards of a systematic review, performing a literature search based on a detailed plan and search strategy, and had the goal of reducing bias by identifying, appraising and synthesising all relevant studies on this topic. In addition, we investigated the specificity of brain correlates. Firstly, we investigated specificity by discussing results from studies using ODD/CD-only samples separately from results from studies using ODD/CD+ADHD samples. Secondly, we compared results of samples including individuals with ODD/CD (with and without comorbid ADHD) to samples including individuals with ADHD-only. In addition, we reported associations between abnormalities in investigated structures and ODD/CD related symptoms. Knowledge about the specificity of reported abnormalities may help to clarify the heterogeneity in studies on ODD/CD.

This review is divided in two sections. The first section deals with structural findings, integrating findings for all reported brain areas; the second section deals with functional findings, and is divided in a part describing hot EF and a subsequent part describing cool EF, based on the assessed tasks and contrasts. Each section starts with a quantitative meta-analysis, for which an activation likelihood estimation (ALE) meta-analysis was performed. ALE is a technique that is used to identify significant anatomical locations for which effects are consistent, and that is robust to publication bias (for a detailed description of ALE see: Eickhoff et al. 2009; Laird et al. 2005; Eickhoff et al. 2012). This quantitative meta-analysis is followed by a narrative review. Then, both the sections on structural and functional findings are integrated to provide a complete overview of all involved brain areas and to assess the evidence for abnormalities in terms of hot and cool EF related brain areas. We expected to find abnormalities in brain structure and function of individuals with ODD/CD subserving both hot EF (i.e., the amygdala, anterior cingulate cortex, insula, and orbitofrontal cortex (Rubia 2011; Prencipe et al. 2011; Crowe and Blair 2008)) and cool EF (i.e. the dorsolateral prefrontal cortex and cerebellum (Rubia 2011; Prencipe et al. 2011; Yang and Raine 2009; Sterzer and Stadler 2009)), which would be in line with an integrated model such as the model proposed by Blair.

## Methods

### Study Selection

This review included all empirical studies that met the following inclusion criteria: (1) the study reported on functional or structural magnetic resonance imaging results, comparing (a) individuals with ODD/CD with or without comorbid ADHD to control subjects, and if included, to individuals with ADHD-only, or (b) individuals with ODD/CD without ADHD to ODD/CD individuals with ADHD. The control group of each study was carefully checked on reported psychiatric disorders, and when a study reported on the presence of any psychiatric disorder in participants of the control group, that study was excluded from the current study. This resulted in the exclusion of two studies. (2) Diagnosis of the participants had to be based on DSM-III, DSM-IV or DSM-5 criteria. (3) The study had to be published in a peer-reviewed English language journal. No limits were set on the ages of participants. All relevant studies published up till June 2015 were incorporated.

**Fig. 1:**
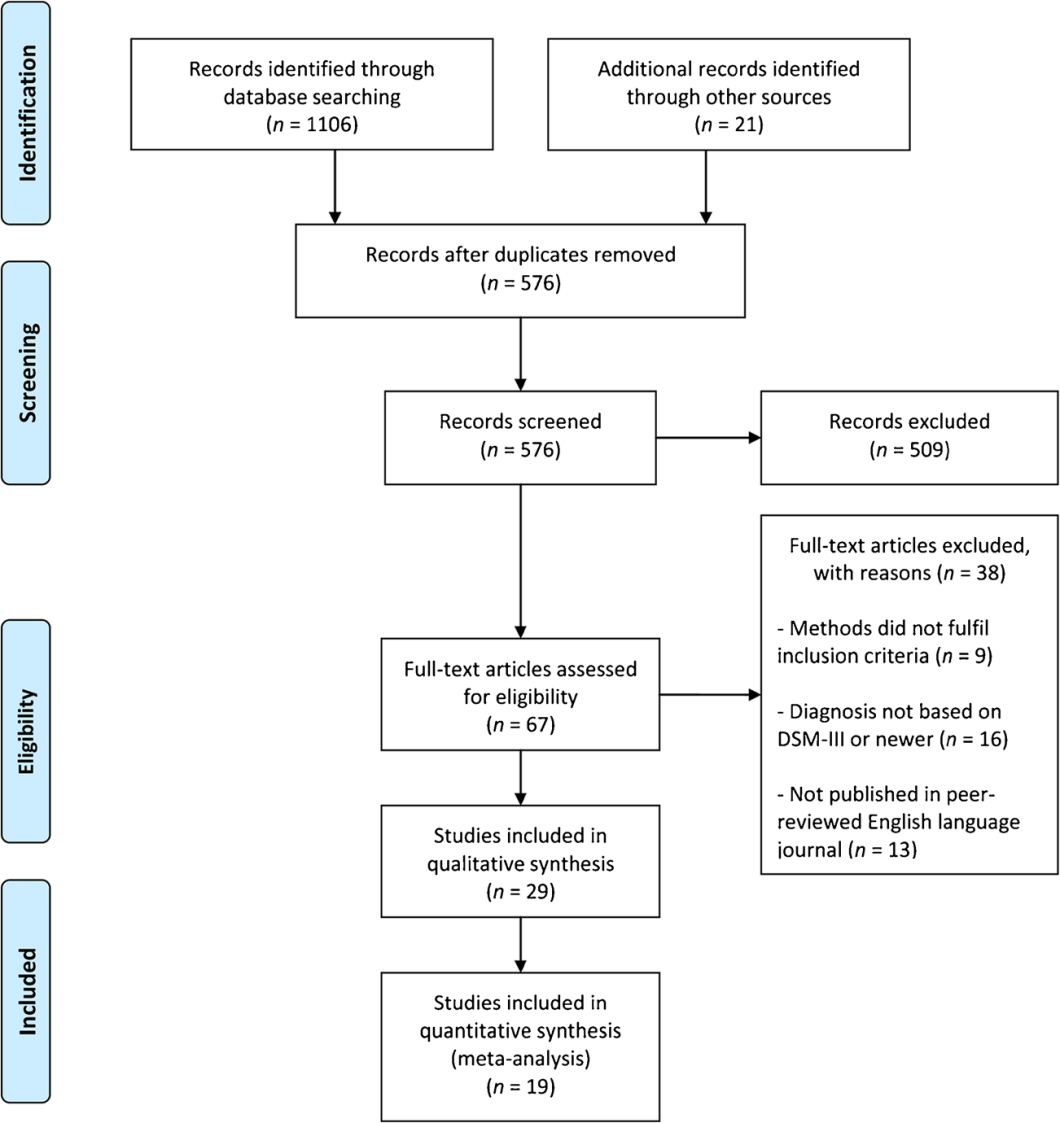
PRISMA flow diagram showing the process of study selection

The databases PubMed, EMBASE, PsycInfo and Web of Science were searched, using the search terms ODD, CD, disruptive behavioural disorder, disruptive behaviour, externalising behavioural disorder, externalising behaviour, MRI, neuroimaging, and equivalent MeSH terms. Furthermore, reference lists of selected studies and reviews were checked for additional relevant studies. A total of 576 studies were initially retrieved and screened, after which a total of 67 studies remained that fulfilled inclusion criteria based on screening of the title and abstract. These 67 studies were further assessed for eligibility using the full text of the study, resulting in 29 studies that met inclusion criteria and were incorporated in the present review; see Fig. 1 for the flow diagram of included studies. The 29 studies selected for review included a total of 1278 individuals, including 713 patients and 565 controls.

In this review, we distinguished the following samples: (1) ODD/CD-only, including only individuals with ODD/CD without comorbid ADHD, (2) ODD/CD-mixed, including both individuals with ODD/CD-only and individuals with ODD/CD+ADHD, and (3) ODD/CD+ADHD, including only individuals classifying for both ODD/CD and ADHD. We included a total of 10 ODD/CD-only studies (7 structural, 3 functional), 14 ODD/CD-mixed studies (9 structural, 5 functional), and 5 ODD/CD+ADHD studies (1 structural, 4 functional). By differentiating between these three samples, it was possible to discern whether an abnormality was related to ODD/CD and not confounded by comorbid ADHD, which would be suggested when an abnormality is found in an ODD/CD-only sample. Alternatively, when an abnormality is found in an ODD/CD-mixed or ODD/CD+ADHD sample, it may be possible that the abnormality is also related to comorbid ADHD. Therefore, results are discussed separately for ODD/CD-only samples versus controls and for ODD/CD-mixed and ODD/CD+ADHD samples versus controls. When included in a study, results from additional comparisons with an ADHD-only sample were provided.

The section on structural neuroimaging findings reports all brain areas, not distinguishing between hot and cool EF. The section on functional neuroimaging findings is divided in two parts: the first part is focused on hot EF and the second part is focused on cool EF. Classification of studies as hot or cool was done in line with the literature. Two authors independently inspected the task characteristics and the reported contrasts for each study and judged these as assessing either hot or cool EF. In case of disagreement, the third author was consulted, resulting in full agreement on all studies. One study (Rubia et al. 2009b) targeted both hot and cool EF and we therefore discussed this study in both parts of the fMRI results. After presentation of the meta-analytic and narrative results of the structural and functional studies, findings are integrated for structural and functional studies in order to provide an overview of all relevant literature and resulting conclusions in terms of hot and cool EF related brain areas.

### Meta-analysis: Activation Likelihood Estimation

For the quantitative meta-analysis, an activation likelihood estimation (ALE) meta-analysis was performed using the Brainmap GingerALE software package (Eickhoff et al. 2009, 2012; Laird et al. 2005). For the ALE meta-analysis to be reliable, the minimum number of studies to be included is five (www.brainmap.org). The algorithm used by GingerALE applies a random-effects approach to identify anatomical locations for which effects are observed most consistently, and which renders it robust for the possible effects of publication bias (Fox et al. 1998). Analyses were performed separately for the structural and the functional studies, and for the functional studies these were performed separately for studies on hot and on cool EF. Studies eligible for inclusion in the meta-analysis were additionally required to report x/y/z coordinates for clusters showing group differences in volume (structural MRI) or activity (functional MRI) in either Montreal Neurological Institute (MNI) or Talairach space.

All coordinates originally reported in MNI space were normalised to Talairach space using Lancaster’s Transform; coordinates which were already in Talairach space were converted back to MNI coordinates and subsequently normalised to Talairach space using Lancaster’s Transform, to account for divergent analyses procedures and minimise differences in coordinates between studies (Lancaster et al. 2007; Laird et al. 2010). The 2.3.1 version of GingerALE (Eickhoff et al. 2009) was applied in the current study and used the coordinates of the reported voxels of each study, referring to the areas that showed a group difference, as a probability distribution to create an ALE distribution map (Turkeltaub et al. 2002; Eickhoff et al. 2009, 2012). Results across studies were aggregated by GingerALE, and modelled activation maps were generated by calculating the probability that a particular voxel was activated as the union of probabilities for that voxel across studies. The contribution of each study to the meta-analytic result was weighted using the study’s sample size, by widening the Gaussian distribution for a voxel with smaller samples to compensate for spatial uncertainty. Meta-analytic maps were generated by combining all the modelled activations maps, and were subsequently corrected for multiple comparisons using Family Wise Error correction. This ensured that differences between studies in terms of the number of areas showing significant group differences (e.g., due to applying a lower statistical threshold), did not influence the combined ALE map (Turkeltaub et al. 2012). This combined ALE map was then compared to a map from a null distribution with the same number of foci, but now randomly placed throughout the grey matter of the brain. After this, the final ALE map was thresholded at *p* < .05 using a False Discovery Rate (FDR) correction for multiple comparisons, and a minimum cluster size of 100 mm^3^ (see www.brainmap.org/ale/).

Both studies using a region of interest (ROI) approach, thus a hypothesis driven pre-selection of specific brain areas, and studies using a whole brain analyses (WBA) approach, thus studying the entire brain, were included in this review. To test the possibility that studies using an ROI approach might bias meta-analytic results due to less stringent statistical criteria, the resulting map of all studies (using both WBA and ROI approach) was compared to the resulting map from studies with a WBA approach. When differences between the maps were present, results from the map including only the WBA studies were reported. The ALE map was overlaid onto a Talairach anatomical template for visualisation purposes, and the areas reported in our Results section refer to the locations of the extrema.

## Results

Results of the reviewed studies are discussed in two main sections with the first section summarising the structural neuroimaging findings and the second section summarising the functional neuroimaging findings. Both sections start with a general overview of all included studies, after which the results from the meta-analysis are reported, followed by the results from the narrative review. The narrative parts contain a summary of the main findings for ODD/CD-only groups first since those are not biased by comorbid ADHD, followed by the main findings in ODD/CD-mixed and ODD/CD+ADHD groups, and ends with the correlational findings and the specificity of findings regarding ODD/CD.

After presentation of the meta-analytic and narrative results of the structural and functional studies, structural and functional findings are integrated in order to provide an overview of all relevant literature and resulting conclusions in terms of hot and cool EF related brain areas. Since an ALE meta-analysis cannot include studies that reported on non-significant group differences, but rather identifies anatomical locations for which effects are observed most consistently, findings from the ALE meta-analysis are combined with the results of the narrative review to provide a comprehensive and balanced overview of available evidence.

### Structural Neuroimaging Findings

#### General Overview

Table 1 shows a total of 12 studies that investigated structural differences in individuals with ODD/CD-only (three studies), ODD/CD-mixed (five studies), or ODD/CD+ADHD (four studies) compared to controls. All 12 studies used WBA to investigate regional brain differences, while three studies additionally used an ROI approach (Fairchild et al. 2011, 2013a; Sterzer et al. 2007). Two of these studies also reported on results of contrasts between ODD/CD (with and without comorbid ADHD) and ADHD-only. In addition, seven studies investigated the association between brain region volumes and symptom counts of ODD/CD (Sterzer et al. 2007; Huebner et al. 2008; Fahim et al. 2011; Fairchild et al. 2011, 2013a; Stevens and Haney-Caron 2012; Michalska et al. 2015).

**Table 1:** Summary of study characteristics and results for structural imaging

Study	Total sample size (% male/ female)	Number of subjects (specified per group)					Age (years, range or M (SD))	Analysis method	Results of comparisons between ODD/CD-only and control group, between ODD/CD+ADHD and control group, and between ODD/CD-mixed and control group. (brain regions reported show reduced volumes in the patient group, unless otherwise reported)			
		ODD/CD -only	ODD/CD+ADHD	ODD/CD-mixed	ADHD- only	Controls			Amygdala	Insula	Prefrontal cortex	Other
1. Fahim et al. 2011 ^a,d^	47^c^ (100 % male)	22	N/A	N/A	N/A	25	Patients: 8.4 (0.10) Controls: 8.4 (0.07)	Whole brain (VBM, cortical thickness) Correlational analyses	*ns*	Bilateral	Left dorsomedial	ODD/CD-only versus controls: VBM: Left medial frontal cortex /claustrum, right inferior frontal cortex/inferior parietal cortex Cortical thickness: left insula, left cingulate, left anterior cingulate, left medial frontal, left rectal/orbitofrontal, left uncus, left precuneus, right middle frontal, right superior temporal, right posterior cingulate ODD/CD-only versus controls: VBM: Negative correlation between right superior temporal cortex, left superior frontal gyrus, right occipital cortex and left precuneus volume and ODD/CD symptom severity
2. Fairchild et al. 2011 ^a,d^	90 (100 % male)	63	N/A	N/A	N/A	27	16–21	ROI; amygdala, insula, anterior cingulate cortex, orbitofrontal cortex (VBM) Whole brain (VBM) Correlational analyses	Bilateral (ROI)	Left (ROI)	Left dorsomedial	ODD/CD-only versus controls: Bilateral caudate, left fusiform gyrus, left inferior and superior occipital cortex ODD/CD-only versus controls: Negative correlation between right insula and CD symptoms
3. Stevens and Haney-Caron 2012 ^a,d^	72 (71 % male)	24	N/A	N/A	24	24	12–18	Whole brain (VBM) Correlational analyses	Left	*ns*	*ns*	ODD/CD-only versus controls: Left inferior frontal, right inferior/middle frontal, right parahippocampal/ fusiform, paracentral cingulate ODD/CD-only versus controls: Positive correlation between bilateral amygdala, bilateral temporal cortex and right lateral orbitofrontal volume and ODD/CD symptom severity
4. Bussing et al. 2002	31 (74 % male)	N/A	12	N/A	N/A	19	8–12	Whole brain (VBM)	*ns*	*ns*	*ns*	ODD/CD+ADHD versus controls: Left and total posterior superior vermis, left and total posterior inferior vermis
5. Kruesi et al. 2004	20^b,c^ (90 % male)	N/A	10	N/A	N/A	10	Patients: 16.1 (3.6) Controls: 15.9 (3.2)	Whole brain (VBM)	*ns*	*ns*	*ns*	ODD/CD+ADHD versus controls: Right temporal lobe
6. Sterzer et al. 2007 ^a,d^	24 (100 % male)	N/A	N/A	12 (5 ODD/ CD-only)	N/A	12	Patients: 12.8 (0.49) Controls: 12.5 (0.45)	ROI: amygdala, anterior insula, anterior cingulate cortex, orbitofrontal cortex (VBM) Whole brain (VBM) Correlational analyses	Left (ROI)	Bilateral (ROI)	*ns*	*ns* ODD/CD-mixed versus controls: Negative correlation between left amygdala and bilateral insula volume and ODD/CD symptom severity (ROI)
7. McAlonan et al. 2007 ^a,d^	59^a,c^ (100 % male)	N/A	28	N/A	N/A	31	6–13	Whole brain (VBM)	*ns*	*ns*	*ns*	ODD/CD+ADHD versus controls: Midline cerebellum, right globus pallidus, right middle frontal gyrus, right superior frontal gyrus, right precuneus, left inferior parietal gyrus, left superior occipital gyrus
8. Huebner et al. 2008 ^a,d^	46^c^ (100 % male)	N/A	N/A	23 (6 ODD/ CD-only)	N/A	23	12–17	Whole brain (VBM) Correlational analyses	Left	*ns*	*ns*	ODD/CD-mixed versus controls: Grey matter, bilateral inferior temporal lobes, left hippocampus, left orbitofrontal gyrus **Increased** bilateral cerebellar volume in ODD/CD-mixed group ODD/CD-mixed versus controls: Negative correlation between bilateral amygdala volume and ODD/CD symptom severity
9. Sasayama et al. 2010 ^a,d^	35 (71 % male)	N/A	10	N/A	8	17	6–16	Whole brain (VBM)	Left	*ns*	*ns*	ODD/CD+ADHD versus controls: Bilateral temporal lobes, bilateral occipital lobes
10. Fairchild et al. 2013a ^,d^	42^b,e^ (100 % female)	N/A	N/A	22 (20 3ODD/ CD-only)	N/A	20	14–20	ROI; amygdala, anterior insula, striatum, anterior cingulate cortex, orbitofrontal cortex (VBM) Whole brain (VBM) Correlational analyses	*ns*	Bilateral (ROI)	Right dorsolateral	ODD/CD-mixed versus controls: Right (ventral) striatum (ROI), right orbitofrontal cortex (ROI), left precentral gyrus, right mid-occipital cortex, right inferior frontal gyrus, left precuneus, middle temporal gyrus ODD/CD-mixed versus controls: Negative correlation between bilateral insula volume and psychopathic traits (ROI), negative correlation between both bilateral insula and left striatum volume and callous-unemotional traits (ROI), positive correlation between bilateral middle/superior orbitofrontal cortex and callous-unemotional traits (ROI).
11. Hummer et al. 2015	66^c^ (73 % male)	14	19	33 (14 ODD/ CD-only)	N/A	33	13–17	Whole brain (VBM)	*ns*	*ns*	*ns*	*ns*
12. Michalska et al. 2015	111^b^ (48 % male)	N/A	43	N/A	N/A	68	9–11	Whole brain (VBM) Correlational analyses	*ns*	*ns*	*ns*	*ns* ODD/CD+ADHD versus controls: Negative correlation between left superior temporal sulcus volume and CD symptoms

Groups did not differ in terms of gender, IQ, or socioeconomic status, unless stated otherwise. *ADHD* Attention Deficit Hyperactivity Disorder; *CD* Conduct Disorder; N/A not applicable; *ns* no significant results are reported for this region; *ODD* Oppositional Defiant Disorder; *ROI* region of interest; *VBM* voxel-based morphometry

^a^Included no data on medication-use or did not adjust for effects of medication-use

^b^Groups differed on IQ, or included no data on IQ

^c^Participants withheld stimulant medication for >24 h prior to scanning

^d^Results included in ALE analysis

^e^Groups differed on socio-economic status

All studies investigated children/adolescents. For information regarding group and study characteristics of the included studies, see Table 1. Reported differences in brain structures are bilateral, unless reported to be either left or right sided. Results are reported from WBA approaches, unless specified to be the result of ROI based analyses, see Table 1.

#### Structural ALE Meta-analysis

Eight structural studies, all using a WBA approach, reported coordinates and were included in the meta-analysis (studies marked with ^a^ in Table 1). Due to the limited number of studies in ODD/CD-only (three studies) it was not possible to perform an ALE meta-analysis on studies comparing ODD/CD-only groups and controls (minimum number of studies required is five). Hence, the ALE meta-analysis was performed on all ODD/CD samples, therewith including both individuals with and without comorbid ADHD. Total sample sizes for all included studies ranged between 24 and 90 individuals, adding up to a total of 415 individuals, of which 267 were patients and 148 were controls (age range 8–21 years). Five studies used a full male sample, two studies used a sample consisting of 71 % males and one study used a full female sample. These studies provided a total of 58 foci of grey matter volume abnormalities in individuals with ODD/CD with and without comorbid ADHD.

**Fig. 2:**
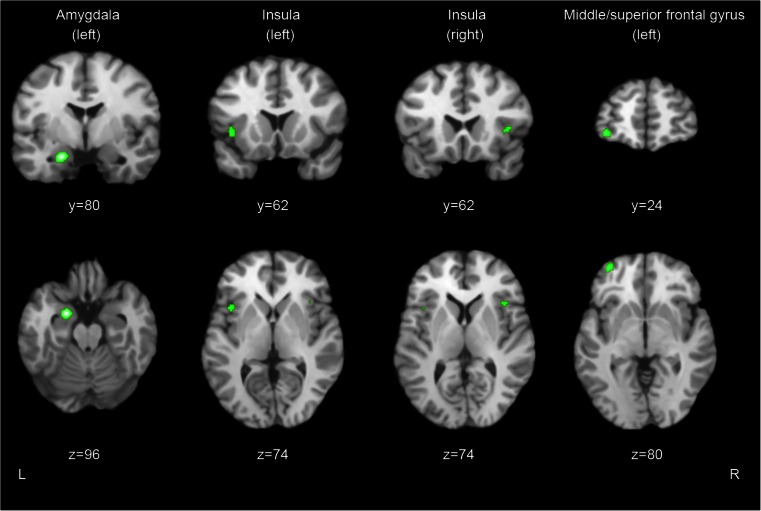
Results of the structural ALE meta-analysis showing the clusters in left amygdala, insula, and left medial/superior frontal gyrus (*p*_corrected_ < 0.05) superimposed on a structural scan in Talairach space. *Top row*: coronal view, *bottom row*: axial view. *L* Left, *R* Right

The ALE analysis revealed four significant clusters of altered grey matter volumes that differed between patients and controls (see Fig. 2 for visualisation). The largest cluster (760 mm^3^) was located in the left amygdala, with three foci inside this cluster. A second cluster (456 mm^3^) was found in the left insula, containing three foci. A third cluster (352 mm^3^) was located in the left medial/superior frontal gyrus, containing two foci. The fourth cluster (216 mm^3^) was located in the right insula, containing two foci.

#### Narrative Review

Table 1 provides an overview of investigated samples and approach (WBA or ROI) and summarizes the findings of the 12 studies reporting on sMRI. Total sample sizes for all included studies ranged between 20 and 111 individuals, adding up to a total of 643 individuals, including 334 patients and 309 controls (age range 6–21 years). The samples consisted predominantly of males, with five studies using a full male sample, six studies using a sample largely consisting of males (ranging from 48 to 90 %), and one study using a full female sample.

*ODD/CD-only* versus *controls (3 studies).* For total grey matter volume, one of three studies reported a reduction in the ODD/CD-only group. In terms of regional grey matter volumes, abnormalities in the insula (left and bilateral), amygdala (left and bilateral), the cingulate cortex (left and paracentral), the inferior frontal gyrus (left and right), and the left dorsomedial prefrontal cortex were repetitively reported (two out of three studies).

*ODD/CD-mixed and ODD/CD+ADHD (9 studies).* Two of the four studies investigating total grey matter in ODD/CD-mixed and ODD/CD+ADHD samples reported smaller volumes in the diagnostic groups, compared to controls. In accordance with results from the studies in the ODD/CD-only group, three of the nine studies in ODD/CD-mixed and ODD/CD+ADHD groups provided support for abnormalities in the left amygdala. Additionally, abnormal volumes were reported in the ODD/CD-mixed and ODD/CD+ADHD groups for structures that were not found for ODD/CD-only groups, which might indicate that these abnormalities reflect the presence of comorbid ADHD rather than ODD/CD. These structures with smaller volumes were the temporal lobe (four out of nine studies: one left, one right, two bilateral) and the cerebellum (three out of nine studies: one left, two bilateral).

*Correlational findings and specificity.* A total of seven studies investigated relations between grey matter abnormalities and ODD/CD symptoms. Three out of seven studies reported associations for the amygdala, insula and temporal cortex. The three studies that reported associations between the amygdala and ODD/CD symptoms, showed either a positive association (thus a larger volume related to more ODD/CD symptoms) for the amygdala in an ODD/CD-only sample, or negative associations (thus a smaller volume related to more ODD/CD symptoms) for the bilateral or left amygdala in ODD/CD-mixed samples. For the insula, all three studies reported negative associations, one between the right insula and ODD/CD symptoms in an ODD/CD-only sample and two between the insula and ODD/CD symptoms in ODD/CD-mixed samples. For the three studies that found associations between the superior temporal cortex and ODD/CD symptoms, one study reported a positive association for the superior temporal cortex in an ODD/CD-only sample, while the other two studies reported a negative association for the right superior temporal cortex in an ODD/CD-only sample and for the left superior temporal cortex in an ODD/CD+ADHD sample.

In terms of specificity of abnormalities for ODD/CD as compared to ADHD, one study reported grey matter reductions in the amygdala and frontal gyrus to be specific for ODD/CD-only, since these were not present in the ADHD-only group. In addition, another study looked at disorder specificity by covarying for ODD/CD in an ODD/CD-mixed group and reported that the grey matter reductions in the amygdala turned non-significant, suggesting that these grey matter reductions were specific for ODD/CD.

#### Structural Summary

Combining the results from the meta-analysis and the narrative review, structural neuroimaging studies most consistently implicated abnormalities of the left amygdala, insula and left frontal gyrus in ODD/CD.

### Functional Neuroimaging Findings

#### General Overview

Tables 2 and 3 shows a total of 17 studies that investigated brain activity correlates in individuals with ODD/CD-only (seven studies), ODD/CD-mixed (nine studies), or ODD/CD+ADHD (one study) compared with typically developing individuals. Of the 17 included studies, 14 conducted a WBA approach to investigate regional brain differences, of which 5 also conducted ROI analyses (S.F. White et al. 2013; Finger et al. 2011; Herpertz et al. 2008; Passamonti et al. 2010; Cohn et al. 2014). In three studies only ROI analyses were reported (Gatzke-Kopp et al. 2009; Marsh et al. 2008; Sterzer et al. 2005). Seven studies investigated the association between activity in brain areas and ODD/CD symptom counts (A.A. Marsh et al. 2013; Sterzer et al. 2005; Passamonti et al. 2010; Cohn et al. 2014; Finger et al. 2008; Rubia et al. 2008, 2010).

**Table 2:** Summary of study characteristics and results for functional imaging – Hot EF

Study	Total sample (% male/ female)	Number of subjects (specified per group)					Age (years, range or M (SD))	Analysis method	Task and conditions	Results of comparisons between ODD/CD-only and control group, between ODD/CD+ADHD and control group, and between ODD/CD-mixed and control group. (brain regions reported show reduced volumes in the patient group, unless otherwise reported)
		ODD/CD- only	ODD/CD+ADHD	ODD/CD -mixed	ADHD- only	Controls				
13. Rubia et al. 2009b ^a,b^	48^c,d^ (100 % male)	14	N/A	N/A	18	16	9–16	Whole brain	Continuous Performance Task - Reward - Non-reward	ODD/CD-only versus controls: Behavioural: No significant group differences, all groups showed equally enhanced performance as a result of reward (versus non-reward trials) *Reward* > *non*-*reward*: reduced activity in right lateral and medial orbitofrontal cortex
14. Kalnin et al. 2011	44^c,d,e^ (59 % male)	22	N/A	N/A	N/A	22	13–17	Whole brain	Emotional Stroop task - Violent words - Non-violent words	ODD/CD-only versus controls: Behavioural: No significant group differences No significant group differences
15. Marsh et al. 2013 ^a^	35^c,d,f^ (66 % male)	14	N/A	N/A	N/A	21	10–17	Whole brain Correlational analyses	Viewing of pictures with three pain intensities (severe/mo-derate/none) during two conditions: pain applied to subject him/herself or to someone else	ODD/CD-only versus controls: Behavioural: No significant group differences *Group differences for the main effect of pain viewing*: reduced activity in left medial frontal gyrus, rostral anterior cingulate cortex, right putamen *Other*’*s pain < own pain:* reduced activity in left amygdala/uncus, left superior frontal gyrus, right insula ODD/CD-only versus controls: Negative correlation between left amygdala activation and aggressive behaviour, negative correlation between left anterior cingulate cortex activation and aggressive behaviour
16. Sterzer et al. 2005	27^d,f^ (100 % male)	N/A	N/A	13 (5 ODD/ CD-only)	N/A	14	9–15	ROI: amygdala, hippocampus, orbitofrontal cortex, anterior cingulate cortex Correlational analyses	Passive viewing task of neutral and negative pictures.	ODD/CD-mixed versus controls: Behavioural: lowered arousal ratings for negative pictures and lowered valence and arousal ratings for neutral pictures in the CD-mixed group compared to controls *Negative > neutral pictures:* reduced activity in left amygdala (ROI), right dorsal anterior cingulate (ROI) ODD/CD-mixed versus controls: Negative correlation between both left amygdala and right anterior cingulate cortex activation and aggressive behaviour (negative versus neutral contrast)
17. Herpertz et al. 2008 ^a^	57^e^ (100 % male)	N/A	N/A	22 (6 ODD/ CD-only)	13	22	12–17	ROI: amygdala, anterior cingulate cortex, orbitofrontal/ medial frontal cortex, insula Whole brain	Passive viewing task Including positive, negative, neutral pictures	ODD/CD-mixed versus controls: Behavioural: CD-mixed evaluated negative and positive pictures as less arousing, and positive pictures as less pleasant compared to controls *Negative > neutral pictures* ***:*** **increased** activity in left amygdala (ROI)
18. Finger et al. 2008 ^a^	42^c,d,e^ (67 % male)	N/A	N/A	14 (4 ODD/ CD-only)	14	14	10–17	Whole brain	Rewarded reversal learning Task - Punished reversal errors - Correct rewarded responses	ODD/CD-mixed versus controls: Behavioural: No significant group differences *Group differences for overall response type (reward and punishment)* ***:*** **increased** activity in left precuneus, right superior frontal gyrus *Punished reversal errors > correct rewarded responses* ***:*** **increased** activity in ventromedial prefrontal cortex, right caudate
19. Marsh et al. 2008	36^e^ (58 % male)	N/A	N/A	12 (5 ODD/ CD-only)	12	12	10–17	ROI: amygdala Correlational analyses	Implicit processing task: categorise gender of fearful, angry, and neutral faces	ODD/CD-mixed versus controls: Behavioural: No significant group differences *Fearful > neutral pictures:* reduced activity in bilateral amygdala (ROI) ODD/CD-mixed versus controls: Negative correlation between connectivity between amygdala and right ventromedial prefrontal cortex, and ODD/CD symptom severity
20. Gatzke-Kopp et al. 2009	30^d,e^ (100 % male)	N/A	N/A	19 (3 ODD/ CD-only)	N/A	11	12–16	ROI: anterior cingulate cortex, caudate, putamen	Monetary incentive task - Reward - Non-reward	ODD/CD-mixed versus controls: Behavioural: No significant group differences *Non-reward > reward:* reduced activity in bilateral anterior cingulate (ROI) **increased** activity in bilateral striatum (caudate) (ROI)
21. Passamonti et al. 2010 ^a^	75 (100 % male)	N/A	N/A	52 (43 ODD/ CD-only)	N/A	23	16–21	ROI: ventromedial prefrontal cortex, amygdala, insula, orbitofrontal cortex Whole brain Correlational analyses	Implicit processing task: categorise gender of angry, sad and neutral faces	ODD/CD-mixed versus controls: Behavioural: No significant group differences *Angry > neutral pictures:* reduced activity in bilateral amygdala (ROI), left insula (ROI),right ventromedial prefrontal cortex (ROI), bilateral dorsolateral prefrontal cortex, bilateral dorsomedial prefrontal cortex, bilateral orbitofrontal cortex (ROI), right inferior parietal cortex, bilateral inferior temporal gyrus, left fusiform gyrus, right middle temporal gyrus, right superior temporal sulcus/gyrus, bilateral thalamus, left putamen, left cerebellum *Sad > neutral pictures:* reduced activity in bilateral amygdala (ROI), ventromedial prefrontal cortex (ROI), left dorsolateral prefrontal cortex, putamen, right cerebellum, bilateral superior temporal sulcus/gyrus ODD/CD-mixed versus controls: Negative correlation between right amygdala and CD symptoms (ROI), negative correlation between ventromedial prefrontal cortex and CD symptoms (ROI), negative correlation between left insula and CD symptoms (ROI)
22. Finger et al. 2011 ^a^	30^f^ (60 % male)	N/A	N/A	15 (5 ODD/ CD-only)	N/A	15	Patients: 14,1 (1,8) Controls: 13,2 (1,1)	ROI: amygdala, orbitofrontal cortex, striatum Whole brain	Passive avoiding learning task - Rewarded correct hits - Punished commission errors	ODD/CD-mixed versus controls: Behavioural: ODD/CD-mixed made more commission errors than controls during the late learning phase (all seven blocks after the first block). *Overall response type (reward and punishment)*: reduced activity in right amygdala (ROI), bilateral superior frontal gyrus, left insula, right medial frontal gyrus, left middle frontal gyrus, left superior parietal lobule, left superior temporal gyrus, left lingual gyrus, right fusiform gyrus, left caudate (ROI), right thalamus *Rewarded correct hits > punished commission errors:* reduced activity in right orbitofrontal cortex (ROI), left middle frontal gyrus, parahippocampal gyrus
23. White et al. 2013 ^a^	38^c,d,f,g^ (Patients: 82 % male Controls: 56 % male)	N/A	N/A	20 (16 ODD/ CD-only)	N/A	18	Patients: 15,2 (2,0) Controls: 14,9 (2,2)	ROI: amygdala, ventromedial prefrontal cortex, caudate Whole brain	Passive avoiding learning task - Reward - Punishment	ODD/CD-mixed versus controls: Behavioural: a smaller proportion of the ODD/CD-mixed group than of the controls showed an association between expected value and choice behaviour *Receiving rewarding feedback modulated by prediction error:* reduced activity in left caudate (ROI) *Receiving punishing feedback modulated by prediction error*: **increased** activity in left caudate (ROI)
24. White et al. 2014 ^a^	30^d,f^ (Patients: 73 % male Controls: 66 % male)	N/A	N/A	15 (8 ODD/ CD-only)	N/A	15	10–17	Whole brain	Reinforcement learning task - Reward (appetitive image) - Punishment (aversive image)	ODD/CD-mixed versus controls: Behavioural: ODD/CD-mixed were less likely to avoid physical threat, but not contamination threat, stimuli than controls *Receiving rewarding feedback modulated by prediction error:* reduced activity in right inferior parietal cortex *Receiving punishing feedback modulated by prediction error*: **increased** activity in right inferior parietal cortex
25. Cohn et al. 2014 ^a^	68 (79 % male)	N/A	N/A	45 (18 ODD/ CD-only)	N/A	23	17,7 (1,6)	ROI: amygdala, ventral striatum, medial prefrontal cortex Whole brain Correlational analyses	Monetary incentive delay task - Reward - Neutral - Loss	ODD/CD-mixed versus controls: Behavioural: No significant group differences *Reward hit > reward miss (reward-feedback):* reduced activity in right ventral striatum (ROI) *Loss miss > hit (loss-feedback):* **increased** activity in right amygdala (ROI) ODD/CD-mixed versus controls: Negative correlation between left amygdala and CU traits (ROI) No correlation between psychopathic traits and neural responses

Participants did not differ in terms of gender, IQ, or socioeconomic status, unless stated otherwise. The italic text describes the contrast for which the results are reported.

*ADHD* Attention Deficit Hyperactivity Disorder; *CD* Conduct Disorder; *N/A* not applicable; *ODD* Oppositional Defiant Disorder; *ROI* region of interest

^a^Results included in ALE

^b^Study included in both hot and cool EF narrative review sections

^c^Groups differed on gender

^d^Groups differed on IQ measures

^e^Participants withheld stimulant medication for >24 h prior to scanning procedure, or did not use medication

^f^Participants did not withheld stimulant medication, but additional analysis did not show an effect of medication use on outcome measures

^g^Groups differed on socio-economic status

**Table 3:** Summary of Study Characteristics and Results for Functional Imaging – Cool EF

Study	Total sample (% male/ female)	Number of subjects (specified per group)					Age (years, range or M (SD))	Analysis method	Task and conditions	Results of comparisons between ODD/CD-only and control group, between ODD/CD+ADHD and control group, and between ODD/CD-mixed and control group. (brain regions reported show reduced volumes in the patient group, unless otherwise reported)
		ODD/CD- only	ODD/CD+ADHD	ODD/CD -mixed	ADHD-only	Controls				
26. Rubia et al. 2008	48–53 (100 % male)	13–14	N/A	N/A	14–20	16–20	9–17	Whole brain	Stop Task - Failed stop - Successful stop - Go	ODD/CD-only versus controls: Behavioural: No significant group differences *Failed stops > go trials:* reduced activity in right posterior cingulate gyrus, right precuneus, left parietal cortex *Go > successful stop trials:* reduced activity in anterior cingulate gyrus, insula, caudate, putamen, thalamus, left superior temporal cortex, premotor cortex
27. Rubia et al. 2009a									Simon Task - Successful congruent - Successful incongruent - Successful oddball	Behavioural:, ODD/CD made more errors compared to controls, no differences between the ADHD-only group and controls *Successful incongruent > successful oddball trials (interference inhibition):* reduced activity in right middle and superior temporal lobe, right precuneus
13. Rubia et al. 2009b ^a^									Continuous Performance - Non-reward - Non-target	Behavioural: No significant group differences *Non-reward > non-target trials (sustained attention):* reduced activity in right insula, right hippocampus, right anterior cingulate cortex, cerebellum, right thalamus, left occipital gyrus, left posterior cingulate, left precuneus
28. Rubia et al. 2010								Correlational analyses (2 out of 4 studies)	Switching Task - Switch - Repeat	Behavioural: No significant group differences, but overall performance was poorer during the switch trials than repeat trials *Group differences for overall task performance:* reduced activity in right inferior parietal lobe, right precentral gyrus, left-superior temporal/inferior parietal cortex, left precuneus, cuneus ODD/CD-only versus controls: Negative correlation between dorsolateral prefrontal cortex and conduct problems (1 study)
29. Zhu et al. 2014	21^b^ (100 % male)	11	N/A	N/A	N/A	10	10–12	Whole brain	GoStop Task - Complete task	ODD/CD-only versus controls: Behavioural: ODD/CD-only showed higher error rate during response inhibition and a longer stop latency *Group differences for overall task performance:* reduced activity in right inferior frontal gyrus, **increased** activity in dorsolateral parts of bilateral inferior frontal frontal gyrus, left middle frontal gyrus, right superior frontal gyrus

Participants did not differ in terms of gender, IQ, or socioeconomic status, unless stated otherwise. The italic text describes the contrast for which the results are reported.

*ADHD* Attention Deficit Hyperactivity Disorder; *CD* Conduct Disorder; *N/A* not applicable; *ODD* Oppositional Defiant Disorder; *ROI* region of interest

^a^Study included in both hot and cool EF narrative review sections

^b^Participants withheld stimulant medication for >24 h prior to scanning procedure, or did not use medication

We reviewed the studies using the framework of hot and cool EF in ODD/CD, which resulted in 13 studies in the hot EF section and 5 studies in the cool EF section, of which 1 study appeared in both sections (Rubia et al. 2009b). The 13 studies in the hot EF section included groups with ODD/CD-only (three studies), ODD/CD-mixed (nine studies) or ODD/CD+ADHD (one study). In all studies, these groups were compared with a typically developing group and in four studies these groups were additionally compared with an ADHD-only group (Finger et al. 2008; Herpertz et al. 2008; Marsh et al. 2008; Rubia et al. 2009b). The five studies in the cool EF section only included individuals with ODD/CD-only that were compared to a typically developing group and in four of the studies the ODD/CD-only group was additionally compared with an ADHD-only group (Rubia et al. 2008, 2009a, b, 2010). Because four of the five cool EF studies were published by the same research group and included largely overlapping samples (Rubia et al. 2008, 2009a, b, 2010), we did not perform an ALE meta-analysis on cool EF fMRI studies in ODD/CD as at least five independent samples are required for this analysis (www.brainmap.org).

All studies investigated children/adolescents. For information regarding group characteristics of the included samples, see Tables 2 and 3. Reported differences in brain structures are bilateral, unless reported to be either left or right sided. Results are reported from WBA approaches, unless specified to be the result of ROI based analyses, see Tables 2 and 3.

#### Hot EF – ALE Meta-analysis

Thirteen functional neuroimaging studies of hot EF were initially included in the meta-analysis. Due to the limited number of studies in ODD/CD-only (three studies) it was not possible to perform an ALE meta-analysis on studies comparing ODD/CD-only groups and controls, thus the ALE meta-analysis was performed on all ODD/CD samples, therewith including both individuals with and without comorbid ADHD.

There was a difference between the resulting maps of the ALE analysis with and without the ROI-only studies. Therefore, we reported the results from the map that included only WBA approach-based studies given the unbiased results. Total sample sizes for all included studies ranged between 30 and 75 individuals, adding up to a total of 423 subjects, of which 256 were patients and 167 were controls (age range 9–21 years). Three studies assessed a full male sample and six studies assessed a sample largely consisting of males (ranging from 56 to 79 %). The studies reported on different tasks associated with hot EF functions: three studies assessed processing of contingencies, two studies assessed passive viewing of emotional pictures, two studies assessed passive avoidance learning, one study assessed active viewing and rating of painful situations, and one study assessed implicit emotional processing.

**Fig. 3:**
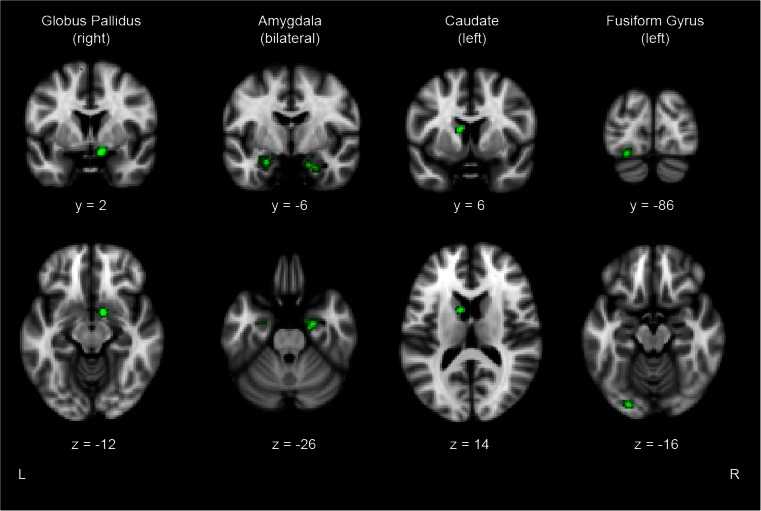
Results of the functional ALE meta-analysis of hot EF showing the clusters in right globus pallidus, bilateral amygdala, left caudate, and left fusiform gyrus (*p*_corrected_ < 0.05) superimposed on a structural scan in Talairach space. *Top row*: coronal view, *bottom row*: axial view. *L* Left, *R* Right

The included studies provided a total of 68 foci of areas showing altered activity in individuals with ODD/CD with and without comorbid ADHD. The ALE analysis revealed five significant clusters representing areas with altered activity in patients with ODD/CD-only or ODD/CD+ADHD that were most consistently reported across the studies (see Fig. 3 for visualisation). The largest cluster (416 mm^3^) was located in the right globus pallidus, with two foci inside this cluster. The second (328 mm^3^) and third (256 mm^3^) clusters were located in the right and left amygdala, and contained three and two foci, respectively. The fourth cluster (208 mm^3^) was present in the left caudate, while the fifth cluster (200 mm^3^) was located in the left fusiform gyrus, both contained two foci.

#### Hot EF – Narrative Review

Tables 2 and 3 provides an overview of investigated samples and approach (WBA or ROI) and summarizes the findings of the 13 included studies reporting on hot EF. Sample sizes ranged between 27 and 75 individuals, adding up to a total of 560 individuals, including 334 patients and 226 controls (age range 9–21 years). The samples consisted predominantly of males, with five studies using a full male sample, and the other eight studies using a sample largely consisting of males (ranging from 56 to 82 %). The studies reported on different tasks associated with hot EF functions: five studies assessed processing of contingencies, three studies assessed passive viewing of emotional pictures, two studies assessed passive avoidance learning, one study assessed active viewing and rating of painful situations, one study assessed implicit emotional processing, and one study assessed interference control with emotional stimuli. Of the 13 studies, 10 used a WBA approach and 3 only an ROI approach, resulting in different numbers of studies per investigated structure.

*ODD/CD-only* versus *controls (3 studies).* Two of the three studies reported abnormalities for ODD/CD-only compared to controls, of which one study reported lower activity in the right striatum, left amygdala, right insula, anterior cingulate cortex, and left medial and superior frontal gyrus, and the other study reported lower activity in the right lateral and medial orbitofrontal cortex.

*ODD/CD-mixed and ODD/CD+ADHD (10 studies).* Studies assessing ODD/CD-mixed and ODD/CD+ADHD groups replicated findings of abnormal function of the striatum (six out of eight studies: three left, two right, one bilateral), including the caudate and putamen, and the amygdala (six out of nine studies: two left, two right, two bilateral). Additionally, functional abnormalities were reported in ODD/CD-mixed groups for the parietal cortex (three out of seven studies: two right, one left), which was not found for ODD/CD-only groups. This might indicate that the abnormal activity in the parietal cortex reflects the presence of comorbid ADHD rather than ODD/CD.

*Correlational findings and specificity.* Five studies investigated relations between activity of specific structures and ODD/CD related symptoms. All five studies reported negative associations (thus a smaller volume related to more ODD/CD symptoms) between amygdala activity and ODD/CD related symptoms, one for an ODD/CD-only sample (left) and four for ODD/CD-mixed samples (two left, one right, one bilateral). Furthermore, two of the five studies reported negative associations between left and right anterior cingulate cortex activity and ODD/CD related symptoms, of which one assessed an ODD/CD-only sample and the other assessed an ODD/CD-mixed sample, respectively.

In terms of specificity, four studies investigated disorder specificity of the abnormalities compared to ADHD-only groups. Of these four studies, one study reported the lower activity in the orbitofrontal cortex in an ODD/CD-only group to be disorder specific, while the other study reported lower activity in the amygdala in an ODD/CD-mixed group to be disorder specific.

#### Hot EF – Summary

Combining the results from the meta-analysis and the narrative review, hot EF functional neuroimaging studies most consistently implicated abnormalities of the amygdala and insula. Even though the meta-analysis implicated the left fusiform gyrus in ODD/CD, the narrative reviews did not show support for abnormalities in this area in ODD/CD.

#### Cool EF – Narrative Review

Tables 2 and 3 provides an overview of investigated samples and approach (WBA or ROI) and summarizes the findings of the five included studies reporting on cool EF. Sample sizes ranged between 21 and 53 individuals, with all studies assessing full male samples (age rang 9–17 years). The studies reported on different tasks associated with cool EF functions: one study assessed inhibitory control, one study assessed interference control, one study assessed attention allocation, one study assessed continuous performance, and one study assessed cognitive flexibility.

*ODD/CD-only* versus *controls (5 studies).* Four of the five studies reported less activation in the precuneus of ODD/CD-only groups, with two studies reporting the left precuneus, one the bilateral precuneus, and one the right precuneus. Furthermore, two of the five studies reported lower activity of the right insula, right anterior cingulate and right posterior cingulate cortex in ODD/CD-only.

*Correlational findings and specificity.* Two studies investigated relations between activity of specific structures and ODD/CD related symptoms, of which one reported a negative association between dorsolateral prefrontal cortex and ODD/CD related symptoms and the other reported no associations. In terms of specificity, four studies investigated disorder specificity of the abnormalities for ODD/CD as compared to ADHD-only. One of these four studies reported abnormal activity in the insula, anterior cingulate cortex, cerebellum and hippocampus to be specific for ODD/CD-only, while another study reported specificity for lower activity in the left parietal-temporal cluster and the right parietal lobe.

#### Cool EF – Summary

The number of available studies into cool EF did not allow a meta-analysis to be conducted. Based on the narrative review, abnormalities in the precuneus were most consistently implicated in ODD/CD.

### Integration of Quantitative and Qualitative Review Findings

In order to determine which hot and cool EF related brain areas are implicated in ODD/CD, findings from both sMRI and fMRI meta-analyses as well as from the narrative reviews are integrated. Since the meta-analyses provide the strongest evidence for the convergence of findings, the main findings of the meta-analyses are discussed first. Second, findings from ODD/CD-only studies (10) are discussed, since these are not biased by comorbid ADHD, followed by findings from the ODD/CD-mixed and ODD/CD+ADHD studies (19). Finally, all these findings are integrated, resulting in an overall conclusion for neuroanatomical abnormalities related to ODD/CD.

Table 4 provides an overview and integration of all reported abnormalities. For the overall conclusion, priority was given to results from the ALE meta-analyses, since these provide the most objective and strong evidence for abnormalities of a certain structure. Qualifications used in the overall conclusion encompassed ‘strong evidence’, ‘some evidence’, ‘weak evidence’ and ‘no evidence’. For the qualification of ‘strong evidence’, the structure had to be reported in both of the ALE meta-analyses. Alternatively, the structure had to be reported in either one of the ALE meta-analyses, while being supported by at least half of the studies included in the narrative reviews (structural and functional) that investigated that structure. For the qualifications of ‘some evidence’ and ‘weak evidence’ at least a quarter of the studies included in the narrative review (structural and functional) that investigated that structure had to report abnormalities, while for ‘some evidence’ the reported abnormality needed additional support of one of the ALE meta-analyses. Finally, when less than a quarter of the studies that investigated that structure reported abnormalities, it was concluded that there was ‘no evidence’ for involvement of that structure in ODD/CD.

**Table 4:** Overview of All Reported Structures and Meta-analytic and Narrative Review Findings

	ALE Meta-Analysis		Abnormalities reported for the studies including samples of ODD/CD-only versus Controls					Abnormalities reported for the studies including samples of ODD/CD-mixed and ODD/CD+ADHD versus Controls					Conclusion ODD/CD versus controls
	Structural	Functional	Structural	Functional		Structural + unctional	Significant associations ^b^	Structural	Functional		Structural + functional	Significant associations ^e^	
				Hot	Cool ^a^				Hot	Cool ^a^			
	(*n* = 8)	(*n* = 9)	(*n* = 3, all WBA)	(*n* = 3, all WBA)	(*n* = 5, all WBA)	(*n* = 11, all WBA)	(*n* = 5)	(*n* = 9, all WBA) ^c,d^	(n varies 7 WBA; 3 ROI) ^d^	(*n* = 0, all WBA)	(n varies) ^d^	(n varies) ^d^	
Total grey matter	NA	NA	1^3^	NA	NA	1	NA	3 / 4^3,5,8^	NA	NA	3 / 4	NA	Some evidence ^f^
Amygdala	Left	Bilateral	2^2,3^	1^15^	*ns*	3	2^3,15^	3^6,8,9^	6 / 9^16,17,19,21,22,25^	NA	9 / 18	5 / 8^6,7,8,19,25^	Strong evidence
Striatum^g^	*ns*	Bilateral	1^2^	1^15^	2^13,26^	4	*ns*	3^1,7,10^	6 / 8^18,20,21,22,23,25^	NA	9 / 17	1 / 5^10^	Strong evidence
Insula	Bilateral	*ns*	2^1,2^	1^15^	2^13,26^	5	1^2^	2^6,10^	2 / 6^21,22^	NA	4 / 15	3 / 6^6,10,21^	Some evidence
Frontal gyrus	Left	*ns*	2^1,3^	1^15^	1^29^	4	1^1^	2^7,10^	2 / 7^18,22^	NA	4 / 16	*ns / 5*	Some evidence
Fusiform gyrus	*ns*	Left	2^2,3^	*ns*	*ns*	2	*ns*	*ns*	2 / 7^21,22^	NA	2 / 16	*ns / 5*	Weak evidence
Temporal cortex	*ns*	*ns*	1^1^	*ns*	2^26,27^	3	2^1,3^	4^5,8,9,10^	2 / 7^21,22^	NA	6 / 16	1 / 5^12^	Weak evidence
(Pre)cuneus	*ns*	*ns*	1^1^	*ns*	4^13,26,27,28^	5	1^1^	2^7,10^	1 / 7^18^	NA	3 / 16	*ns / 5*	Weak evidence
Parietal cortex	*ns*	*ns*	1^1^	*ns*	2^26,28^	3	*ns*	1 ^7^	3 / 7^21,22,24^	NA	4 / 16	*ns / 5*	Weak evidence
Anterior cingulate	*ns*	*ns*	*ns*	*ns*	2^13,26^	2	1^15^	2^1,3^	3 / 6^15,16,20^	NA	5 / 16	1 / 7^16^	Weak evidence
Orbitofrontal cortex	*ns*	*ns*	1^1^	1^13^	*ns*	2	*ns*	2^8,10^	2 / 8^21,22^	NA	4 / 17	*ns / 7*	No evidence
Prefrontal cortex	*ns*	*ns*	2^1,2^	*ns*	*ns*	2	1^28^	1^10^	2 / 7^18,21^	NA	3 / 16	2 / 6^19,21^	No evidence
Cerebellum	*ns*	*ns*	*ns*	*ns*	1^13^	1	*ns*	3^4,7,8^	1 / 7^21^	NA	4 / 16	*ns / 5*	No evidence
Occipital cortex	*ns*	*ns*	1^2^	*ns*	1^13^	2	*ns*	3^7,8,10^	*ns / 7*	NA	3 / 16	*ns / 5*	No evidence
Cingulate	*ns*	*ns*	2^1,3^	*ns*	2^13,26^	4	*ns*	1^15^	*ns / 7*	NA	1 / 16	*ns / 5*	No evidence
Hippocampus	*ns*	*ns*	*ns*	*ns*	1^13^	1	*ns*	1^8^	1 / 8^22^	NA	2 / 17	*ns / 5*	No evidence
Pre-central gyrus	*ns*	*ns*	*ns*	*ns*	1^28^	1	*ns*	1^10^	*ns / 7*	NA	1 / 16	*ns / 5*	No evidence
Thalamus	*ns*	*ns*	*ns*	*ns*	2^13,26^	2	*ns*	*ns*	1 / 7^21^	NA	1 / 16	*ns / 5*	No evidence
Lingual gyrus	*ns*	*ns*	*ns*	*ns*	*ns*	*ns*	*ns*	*ns*	1 / 7^22^	NA	1 / 16	*ns / 5*	No evidence
Uncus	*ns*	*ns*	1^1^	*ns*	*ns*	1	*ns*	*ns*	*ns / 7*	NA	1 / 16	*ns / 5*	No evidence
Premotor cortex	*ns*	*ns*	*ns*	*ns*	1^13^	1	*ns*	*ns*	*ns / 7*	NA	1 / 16	*ns / 5*	No evidence

First column with results shows the meta-analytical results, second column shows results from studies using ODD/CD-only samples, third column shows results from studies using ODD/CD-mixed and ODD/CD+ADHD samples, last column shows overall conclusion. For the second and third columns the numbers in the columns refer to the numbers of studies showing significant results for this structure, numbers in superscript refer to the reviewed studies in Table 1 (structural studies) or Tables 2 and 3 (functional studies). For the overall conclusion on involvement of structures in ODD/CD, three qualifications were used: strong evidence, some evidence, weak evidence, and no evidence. The qualification strong evidence was used if a structure was found involved in ODD/CD (1) in both meta-analyses OR (2) either one of the meta-analyses AND by half or more of the studies in the narrative review. The qualification some evidence was used if a structure was found involved in ODD/CD in either one of the meta-analyses AND by a quarter or more of the studies in the narrative review. The qualification weak evidence was used if a structure was not found involved in ODD/CD in either one of the meta-analysis, but was found in a quarter or more of the studies in the narrative review. The qualification no evidence was used if a structure was not found involved in ODD/CD in either one of the meta-analysis, and additionally was found by less than a quarter of the studies in the narrative review. *ADHD* Attention Deficit Hyperactivity Disorder; *CD* Conduct Disorder; *NA* not applicable; *ns* not significant; *ODD* Oppositional Defiant Disorder; *ROI* region of interest; *WBA* whole brain analysis

^a^Four of the five studies pertained to largely overlapping samples

^b^Two structural, one hot EF and two cool EF studies

^c^For the studies using ODD/CD-mixed and ODD/CD+ADHD samples, only four of the structural studies investigated total grey matter

^d^Results are given according to the format ‘number of studies reporting significant findings for this structure / total number of studies reporting on this structure’

^e^Six structural, five hot EF and two cool EF studies

^f^The qualification ‘some evidence’ was given although there were no meta-analytical findings, since a meta-analysis was not possible for total grey matter

^g^Including caudate, putamen, globus pallidus

#### Meta-analyses

Meta-analytic results implicated both structural and functional abnormalities for one of the hot EF related structures: the amygdala (left and bilateral, respectively). Other hot EF related areas were only implicated in the structural (insula, left frontal gyrus) or functional meta-analyses (striatum, left fusiform gyrus).

#### Narrative Review: ODD/CD-Only Versus Controls (10 Studies)

Integration of the structural and functional findings implicated abnormalities in several hot EF related structures for ODD/CD, since these structures were reported in more than a quarter of the studies: the amygdala, right striatum, insula, and left frontal gyrus (see Table 4). For a range of structures there was only weak or no evidence for abnormalities in ODD/CD (see Table 4 for an overview).

#### Narrative Review: ODD/CD-Mixed and ODD/CD+ADHD (19 Studies)

Overall, findings from the ODD/CD-mixed and ODD/CD+ADHD groups again showed abnormalities in several hot EF related structures for ODD/CD: the amygdala, right striatum, insula and left frontal gyrus (see Table 4). For the amygdala and right striatum at least half of the structural and functional studies investigating those structures reported abnormalities, and for the insula and left frontal gyrus more than a quarter of the structural and functional studies investigating those structures reported abnormalities. Again, there was only weak or no evidence for involvement of a range of structures (see Table 4 for an overview).

#### Overall Conclusion and Specificity of Findings

Integration of the structural and functional findings across the ODD/CD-only, ODD/CD-mixed and ODD/CD+ADHD samples provided strong evidence for abnormalities in structure and function of two hot EF related structures: the amygdala and the striatum (see Table 4). Additionally, there was some evidence for structural and functional abnormalities in two other hot EF related structures: the insula and the left frontal gyrus (see Table 4).

Further support for involvement of abnormalities of some of these structures in ODD/CD was established by comparing the results from studies using ODD/CD-only samples with the results from studies using ODD/CD-mixed and ODD/CD+ADHD samples to see if results were mainly driven by ODD/CD, and reviewing the correlational and specificity findings. The involvement of the amygdala in ODD/CD was further supported by the finding that abnormalities were mainly driven by the studies including ODD/CD-only or ODD/CD-mixed groups, and to a lesser extent by studies including ODD/CD+ADHD groups. Hence, it seems that abnormalities are related to ODD/CD and not to comorbid ADHD. Furthermore, there was strong evidence from studies on specificity of abnormalities in the amygdala for ODD/CD as compared to ADHD-only, and there was strong evidence for an association between both amygdala structure and function and ODD/CD related symptoms. Similar evidence was reported for the insula, where again the results were mainly driven by studies including ODD/CD-only or ODD/CD-mixed groups, rather than by studies including ODD/CD+ADHD groups. Also, some evidence for an association between both insula structure and function and ODD/CD related symptoms was present. For the striatum there was some evidence for an association between abnormalities in this area and ODD/CD related symptoms. For the other areas (frontal and fusiform gyrus) there was no further supporting evidence from studies investigating specificity by comparing an ODD/CD-only sample with an ADHD-only sample, nor did correlational approaches provide evidence for associations between abnormalities in these areas and ODD/CD symptoms.

Finally, driven by results from the cool EF section using only ODD/CD-only samples, some evidence was found for abnormalities in a cool EF related structure in ODD/CD: the left precuneus. However, studies that compared ODD/CD-only samples with ADHD-only samples did not provide evidence for these abnormalities to be specific for ODD/CD, nor did studies that assessed associations provide evidence for precuneus volume to be related to ODD/CD symptoms. Taken together, our results show strong evidence for involvement of the amygdala and the striatum, some evidence for involvement of the insula and the frontal gyrus, and weak evidence for involvement of the precuneus in ODD/CD.

## Discussion

We reviewed 29 structural and functional neuroimaging studies into hot and cool EF in ODD/CD (with and without ADHD) and performed two meta-analyses on subsets of these studies. We hypothesized that, compared to controls, individuals with ODD/CD (with and without ADHD) would show both structural and functional brain abnormalities in areas related to hot and cool EF. In addition, we investigated the specificity of these abnormalities by discussing results separately for studies including an ODD/CD-only group and results from studies including an ODD/CD-mixed or ODD/CD+ADHD group, using a narrative approach. Furthermore, individuals with ODD/CD were compared to individuals with ADHD. The results confirmed impairments in structure and function in most of the hypothesised hot EF related structures (i.e. amygdala, insula, and anterior cingulate), and to a lesser extent results confirmed such impairments in cool EF related structures (i.e. dorsolateral prefrontal cortex and its subcortical connections, including the cerebellum). Impairments were mainly present as reductions in volume or reductions in activity of the structures. Some of these abnormalities were reported to be specific to ODD/CD when compared to ADHD-only, or showed associations with ODD/CD symptom levels.

In terms of hot EF related neuroanatomical correlates, several structures were consistently reported to be associated with ODD/CD (with and without ADHD) compared to controls (see Table 4 for an overview). This pattern of findings emerged irrespective of a structural or functional approach of the study. For two areas the combination of the narrative review and meta-analyses of both structural and functional MRI studies provided strong evidence of abnormalities in ODD/CD. These areas were the bilateral amygdala and the right striatum, including the caudate, putamen, and globus pallidus. In addition to these two areas, some evidence was provided for abnormalities of the bilateral insula in ODD/CD groups. For all three areas, the majority of studies reported reductions in volume and activity in ODD/CD with and without ADHD groups compared to controls. Furthermore, for all three areas abnormalities appeared to be mainly driven by studies including ODD/CD-only or ODD/CD-mixed groups, and to a lesser extent by studies including ODD/CD+ADHD groups. Hence, it seems that ODD/CD may drive these abnormalities, rather than ADHD. For the amygdala, this claim is supported by strong evidence for the abnormalities to be specific for ODD/CD when compared to ADHD-only and by a significant association between abnormalities in the amygdala and ODD/CD symptoms in both ODD/CD-only and ODD/CD-mixed groups. For the insula there was some evidence for specificity for ODD/CD compared to ADHD and some evidence for an association with ODD/CD symptoms. However, for the right striatum there was neither evidence for specificity for ODD/CD nor for an association between ODD/CD related symptoms.

The finding of abnormalities in brain areas related to hot EF for ODD/CD is in line with the abnormalities observed in ODD/CD in terms of performance on hot EF related tasks. The amygdala, striatum, insula, and frontal gyrus are implicated in emotion processing (Schumann et al. 2011; Anderson and Kiehl 2012), reinforcement processing (Di Martino et al. 2008; Helie et al. 2013; Jessup and O’Doherty 2011), empathy (Gu et al. 2012, 2013; Lamm and Singer 2010), and introspection (Passamonti et al. 2012; Goldberg et al. 2006) in typically developing controls, respectively. There is robust support for abnormalities in emotion processing, altered reinforcement sensitivity, deficits in empathy and abnormalities in self-control in individuals with ODD/CD (Blair 2013; Byrd et al. 2014; Burke et al. 2002).

In terms of cool EF related neuroanatomical correlates, few significant findings emerged. Partly, this was due to the limited number of functional neuroimaging studies into cool EF in individuals with ODD/CD. Almost all available functional studies were performed within the same sample, precluding a meta-analysis of these findings and restricting our approach to a narrative review of studies. For the left frontal gyrus some evidence for abnormalities in ODD/CD was found. For the left precuneus weak evidence was found, however this structure was predominantly reported in studies using ODD/CD-only groups, suggesting involvement of this structure in ODD/CD. Future studies should confirm this claim. Surprisingly, there was no evidence for involvement of abnormalities of more typical cool EF structures, such as the dorsolateral prefrontal cortex or the cerebellum in ODD/CD. The lack of robust cerebellum-related results in ODD/CD may be due to abnormalities in the cerebellum being more strongly related to comorbid ADHD, than to ODD/CD itself. Some support for this interpretation was found, since structural and functional abnormalities in the cerebellum were reported mainly in studies including ODD/CD+ADHD and ODD/CD-mixed groups, and not so much in studies including ODD/CD-only groups.

The absence of a significant relationship between brain areas related to cool EF and ODD/CD is in line with the inconsistent findings on tasks assessing cool EF in this group. ODD/CD seems to be associated with specific inhibitory-related abnormalities (Willoughby et al. 2011; Giedd et al. 2010; Hobson et al. 2011), rather than with more general cool EF abnormalities. This is emphasised by results from studies in other cool EF related domains, such as cognitive flexibility and working memory, that showed these domains to be intact in ODD/CD and to be associated with comorbid ADHD instead of ODD/CD (Dawel et al. 2012; Giedd et al. 2010; Hobson et al. 2011; Prencipe et al. 2011).

**Fig. 4:**
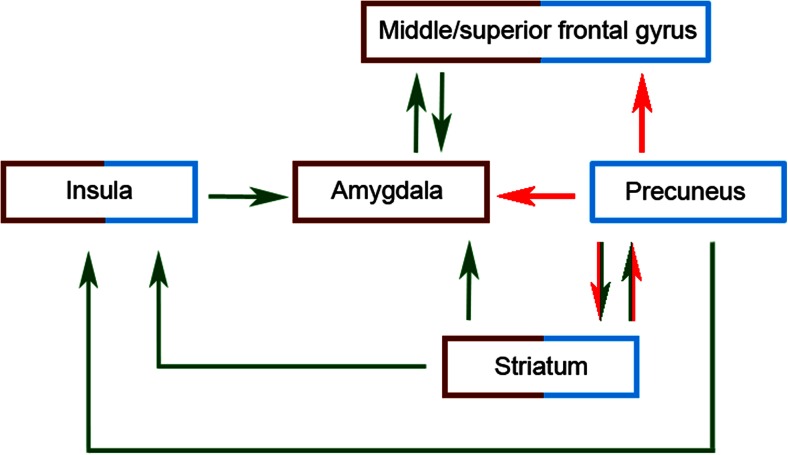
Overview of the connectivity of areas involved in ODD/CD described in this review, based on connectivity studies in typically developing individuals (see main text). Green arrows indicate excitatory connectivity, red arrows indicate inhibitory connectivity, and arrows in both green and red indicate both excitatory and inhibitor connectivity. Red boxed areas exhibit mainly hot EF, blue boxed areas exhibit mainly cool EF, and areas boxed in both red and blue exhibit a combination of hot and cool EF

Although we have suggested that abnormalities in ODD/CD are mainly associated with hot EF, most of the reported hot EF brain areas are also involved in cool EF (see Fig. 4 for visualisation), implying an integrated model of abnormalities in both hot and cool EF, rather than a strict separation between these two. This is in line with models that propose abnormalities in both hot and cool EF circuits to underlie the behavioural characteristics of ODD/CD, such as the model by Blair (2005). The connectivity between the structures we found to be involved in ODD/CD (amygdala, insula, striatum, medial/frontal gyrus, precuneus) has been extensively studied, and studies show that all these areas appear to be interconnected, albeit each to a different degree, and are found to subserve several different functions. For example, it is well established that the amygdala, as part of the limbic system, is critical for hot EF, such as the processing of emotional information. Moreover, it also modulates behavioural responses subsequent to emotionally salient information, with the regulatory process being regarded as a cool EF related process (Schumann et al. 2011; Anderson and Kiehl 2012). The insula is also involved in emotional information processing, and is necessary for empathic pain perception and compassion, hence referring to hot EF, but also for the integration of emotional and cognitive processes, thus referring to cool EF (Gu et al. 2012, 2013; Lamm and Singer 2010). The striatum, specifically the dorsal part (mainly caudate and putamen), is associated with inhibitory functions as well as with reinforcement learning, and thus referring to both cool and hot EF related processes, respectively (Di Martino et al. 2008; Helie et al. 2013; Jessup and O’Doherty 2011). The medial/superior frontal gyrus is associated with response inhibition and other cool EF functions, but also with introspection, which can be seen as a more hot EF related process (Passamonti et al. 2012; Goldberg et al. 2006). The precuneus, finally, is an area that is involved in reflective, self-related processing and awareness, incorporating both hot and cool EF processes (S. Zhang and Li 2012). Overall, it shows that our findings of abnormalities in the five abovementioned structures may lead to impairments that show striking parallels with the behavioural characteristics of individuals with ODD/CD, such as their difficulties in learning socially acceptable behaviour, and their tendency to contribute hostile intentions to peers in socially ambiguous situations (Byrd et al. 2014; Blair 2013; Burke et al. 2002). This supports an integrated model of hot and cool EF abnormalities in ODD/CD, for which the model by Blair (2005) is currently the most comprehensive and encompasses a full description of both circuits.

Our results regarding abnormalities in brain structure and function in ODD/CD are in line with models on brain functioning of other externalising disorders for which ODD and CD are risk-factors, such as antisocial personality disorder (ASPD) or psychopathy (American Psychiatric Association 2013). ASPD and psychopathy are characterised by symptoms of emotional detachment and impulsive behaviour, and the disregard for, or violation of, rights of others. The overlap in neurocognitive and behavioural abnormalities between ASPD, psychopathy and ODD/CD, suggests that these disorders may share an underlying aetiology. A recently proposed neurocognitive model of psychopathy includes the paralimbic system as a whole, comprising both the frontal lobe and the temporal lobe (including the amygdala). A review on psychopathy reported that most consistently implicated areas were the amygdala, anterior and posterior cingulate cortices, orbitofrontal cortex and adjacent (para)limbic structures (Yang and Raine 2009). Furthermore, these brain areas are also implicated in ASPD (Kiehl 2006; Blair 2010). Since we found some of these areas to be involved in ODD/CD, a shared aetiology with ASPD and psychopathy seems plausible.

### Research Agenda

The goal of the current review was to aggregate and integrate findings from structural and functional studies on brain anatomical aspects of ODD/CD, to provide a complete overview of brain abnormalities associated with ODD/CD. During this process we encountered several issues in the field. Firstly, in our meta-analysis of hot EF functional studies, different results were obtained when the analyses were conducted with and without inclusion of ROI-based studies, suggesting that ROI-based studies may bias conclusions. We therefore restricted our hot EF meta-analysis to WBA approach-based studies, given their unbiased results. Future studies investigating brain abnormalities associated with ODD/CD should not only include ROI analyses, but would benefit from reporting (additional) whole brain analyses, rendering the results eligible to be included in a meta-analysis. In addition, because of the limited amount of studies into cool EF and the overlap in subjects in these studies, we were not able to perform a meta-analysis on these data; further research is needed in order to be able to draw firm conclusions on the possible involvement of cool EF abnormalities in ODD/CD, particularly since evidence from behavioural studies into cool EF impairments in ODD/CD is inconclusive.

Secondly, in terms of sample characteristics, an important future direction is to study the role of callous-unemotional (CU) traits or psychopathic traits in individuals with ODD/CD. It has been reported that children with CD and high levels of CU traits show greater abnormalities in reward sensitivity, lower responsivity to treatment by parenting strategies, and are more resistant to psychosocial intervention than those with CD and few CU traits (Byrd et al. 2014). Another important issue in terms of the investigated samples is that most of the studies focussed on males, limiting generalization of current findings. This is emphasised by the differing pathways of brain development in boys and girls (Haney-Caron et al. 2014), and the fact that boys and girls seem to differ in the neural characteristics of ODD/CD (Hyatt et al. 2012). For example, when comparing male and female samples with ODD/CD-mixed, gender differences emerged for the insula (Fairchild et al. 2013a). Moreover, since substantial developmental changes take place during the transition from childhood to adulthood (Giedd et al. 2009), studies should take developmental patterns into account. This can be achieved by adhering to smaller age ranges when including participants, but also by matching on age or using large samples that allow developmental patterns to be studied. In the majority of the included studies, participant ages ranged between 10 and 17 years, which cover a substantial time of brain development. Furthermore, since ODD and CD are childhood disorders and are referred to as APD when persisting into adulthood, our findings may not be generalizable to older individuals.

Thirdly, at a statistical level, future studies should be more consistent in statistical corrections and thresholding. A significant proportion of the studies did not correct for multiple comparisons or Type 1 errors, nor applied cluster thresholding. Of these, most studies using a WBA approach did report Family Wise Error or False Discovery Rate corrected results, except for three structural and four functional studies (Bussing et al. 2002; Kruesi et al. 2004; Fairchild et al. 2013a; Marsh et al. 2013; Passamonti et al. 2010; White et al. 2013, 2014). However, most of these studies did set a minimum number of >10 contiguous voxels and lowered the statistical threshold to *p* < .005 (Marsh et al. 2013; White et al. 2013, 2014) or *p* < .001 (Passamonti et al. 2012; Fairchild et al. 2013a), leaving only two studies that reported at an uncorrected statistical threshold of *p* < .05. This implies that the results of both the meta-analyses and narrative reviews are in general reliable, but should be interpreted with some caution, since especially in WBA-based studies, these statistical methods are of importance (Chumbley and Friston 2009; Chumbley et al. 2010; Nichols and Hayasaka 2003).

Fourthly, future studies should look at other imaging techniques, such as DTI, to improve our knowledge on structural and functional brain abnormalities associated with ODD/CD. Up to date, eight studies have used DTI to study abnormalities in pathways in ODD/CD. Although there is relatively consistent evidence for involvement of the uncinate fasciculus (Wang et al. 2012; Finger et al. 2012; Haney-Caron et al. 2014; Passamonti et al. 2012; Sarkar et al. 2013; Zhang et al. 2014a), studies largely differed in terms of the white matter structures implicated in ODD/CD. Furthermore, while some studies report heightened DTI measures in ODD/CD (Passamonti et al. 2012; Sarkar et al. 2013; Zhang et al. 2014a, 2014b), other studies report lowered DTI measures in ODD/CD (Haney-Caron et al. 2014; Wang et al. 2012), or no differences at all (Finger et al. 2012). In addition to integrating findings of DTI studies, future studies could look at other imaging techniques, such as resting-state fMRI and combined sMRI-fMRI studies.

Finally, to advance our understanding of ODD and CD and the specificity of neural abnormalities future studies should assess well-defined and sufficiently sized samples. For example, the ideal study should at least compare ODD/CD-only, ADHD-only, comorbid ODD/CD+ADHD, and typically developing control groups with sufficient sample size, to investigate the possible confounding effect of comorbid ADHD. Furthermore, since there appears to be a difference in underlying mechanisms for childhood-onset and adolescent-limited ODD/CD (Moffitt 1993), future studies should differentiate between these types of ODD/CD. Moreover, studies should include separate groups of individuals with pure ODD and pure CD, to investigate whether these disorders are indeed two ends of a spectrum or rather two separate disorders, since this is not completely evident from studies performed so far (Matthys et al. 2013; Rowe et al. 2010). A strict separation of the two disorders was not possible at the time of the current review, given that until the release of the DSM-5, a diagnosis of ODD was precluded when CD was present. Therefore most studies into CD did not investigate or report on ODD, possibly limiting the generalization of our findings to ODD-only and CD-only samples. In terms of ADHD comorbidity, most studies that reported on ODD/CD did not investigate (or report on) possible comorbidity with ADHD, hence, studies investigating ODD/CD-only samples are sparse. For this reason, we could not perform a meta-analysis on studies that compared groups with ODD/CD-only to groups with ADHD-only. Also, it would be of great interest to investigate whether ADHD comorbidity adds abnormalities associated with ADHD to those already present due to ODD/CD, or that ODD/CD with comorbid ADHD is a genuinely separate disorder. Furthermore, in the current review we did not examine the impact of other common comorbidities such as anxiety or depression. This is especially important because these comorbidities are associated with abnormalities in some of the same brain areas that appear to be implicated in ODD/CD (Sterzer et al. 2005). Especially the amygdala is related to both anxiety and depressive disorder, albeit in an opposite direction, showing higher volumes, instead of the lower volumes, to be associated with ODD/CD (Machado-de-Sousa et al. 2014; Young et al. 2014). Furthermore, not all studies investigated (or reported on) (past) medication use, even though in almost all studies, MRI scanning was done in individuals free of medication at the time of testing (Rubia et al. 2013). Current and past mediation use should be incorporated in future studies, since this may have long-term effects on brain structure and function (Hart et al. 2013; Rubia et al. 2014).

## Conclusion

We reviewed 29 studies on structural and functional neuroimaging in samples with ODD/CD with and without comorbid ADHD, and performed meta-analyses on a subset of eight structural studies and nine hot EF functional studies. Despite some limitations and heterogeneity amongst studies, results indicated that individuals with ODD/CD primarily show abnormalities in the bilateral amygdala, bilateral insula, right striatum and left medial/superior frontal gyrus as well as the left precuneus. Evidence of involvement of these areas was present in both structural and functional studies, and irrespective of whether the study included individuals with ODD/CD-only or with ODD/CD with comorbid ADHD. Our results show strong evidence of specificity for abnormalities in the amygdala for ODD/CD as compared to ADHD, and correlational studies further support the association between abnormalities in the amygdala and ODD/CD symptoms. Besides the left precuneus that was revealed by the narrative review of cool EF, there was no evidence for abnormalities in typical cool EF related structures, such as the cerebellum and dorsolateral prefrontal cortex. Our findings confirm the involvement of hot, and to a smaller extent cool EF associated brain areas in ODD/CD, and support an integrated model of both hot and cool deficits for ODD/CD (e.g. Blair 2005). The areas found associated with ODD/CD are involved in emotion-processing, error monitoring, self-control, and empathic and social behaviour. It is precisely these functions that are impaired in children and adolescents with ODD/CD, and that result in difficulties learning socially accepted behaviours and reactions, attributing hostile intentions to others, and preferring aggressive solutions to social dilemmas.
